# Incidence and Outcomes of Anterior Chamber Gas Bubble during Femtosecond Flap Creation for Laser-Assisted In Situ Keratomileusis

**DOI:** 10.1155/2015/542127

**Published:** 2015-04-20

**Authors:** Sloan W. Rush, Philip Cofoid, Ryan B. Rush

**Affiliations:** ^1^Panhandle Eye Group, 7400 Fleming Avenue, Amarillo, TX 79106, USA; ^2^Texas Tech University Health Sciences Center, 1400 S. Coulter, Amarillo, TX 79106, USA; ^3^Southwest Retina Specialists, 7411 Wallace Boulevard, Amarillo, TX 79106, USA

## Abstract

*Purpose*. To report the incidence and outcomes of anterior chamber gas bubble formation during femtosecond laser flap creation for laser-assisted in situ keratomileusis (LASIK). *Methods*. The charts of 2,886 consecutive eyes that underwent femtosecond LASIK from May 2011 through August 2014 were retrospectively reviewed. The incidence, preoperative characteristics, intraoperative details, and postoperative outcomes were analyzed in subjects developing anterior chamber gas bubble formation during the procedure. *Results*. A total of 4 cases (0.14%) developed anterior chamber gas bubble formation during femtosecond laser flap creation. In all four cases, the excimer laser was unable to successfully track the pupil immediately following the anterior chamber bubble formation, temporarily postponing the completion of the procedure. There was an ethnicity predilection of anterior chamber gas formation toward Asians (*p* = 0.0055). An uncorrected visual acuity of 20/20 was ultimately achieved in all four cases without further complications. *Conclusions*. Anterior chamber gas bubble formation during femtosecond laser flap creation for LASIK is an uncommon event that typically results in a delay in treatment completion; nevertheless, it does influence final positive visual outcome.

## 1. Introduction

Cavitation gas bubbles are an expected phenomenon during femtosecond laser flap creation for laser-assisted in situ keratomileusis (LASIK) [1, 2]. An opaque bubble layer (OBL) is a well-known intraoperative finding on various femtosecond laser platforms [3, 4]. In rare instances, the OBL may temporarily preclude pupillary tracking during the excimer laser portion of the femtosecond LASIK procedure [5]. Bubbles that are confined to the corneal stromal bed may disperse rapidly, and there are manual surgical techniques that may expedite their dissipation [6]. Some femtosecond laser platforms have designed a venting canal incision at the hinge of the flap to facilitate the release of the cavitation bubbles external to the lamellar cutting plane in order to negate the formation of a OBL [7]. In contrast to the OBL in the corneal stroma, there have been only a few case reports in which there was formation of a gas bubble inside the anterior chamber [8, 9]. By comparison, anterior chamber gas bubbles may not absorb as promptly as stromal bed OBL and can potentially inhibit the excimer laser from adequately tracking the pupil [10]. Various mechanisms have been hypothesized to describe the occurrence of anterior chamber gas bubbles [11, 12], but little is known regarding their incidence, risk factors, clinical significance, and intraoperative/postoperative consequences. In this study, we describe the incidence, baseline characteristics, and postoperative outcomes in subjects developing anterior chamber gas bubble formation during femtosecond laser flap creation during LASIK.

## 2. Methods

An institutional review board [13] approved this retrospective, consecutive chart review that included all patients from May 2011 through August 2014 that received femtosecond laser flap creation for LASIK at a single center, Rush Eye Associates, located in Amarillo, TX, USA. All research components adhered to the tenets of the Declaration of Helsinki and were conducted in accordance with human research regulations and standards.

### 2.1. Inclusion/Exclusion Criteria and Data Collection

The operative eyes of all patients that underwent femtosecond LASIK on the Wavelight FS200 femtosecond laser and the Allegretto Wave Eye-Q 400 Hz excimer laser platforms (Alcon, Fort Worth, TX, USA) by a single surgeon (SWR) during the aforementioned study interval were included. For all cases in which anterior chamber gas bubble formation occurred, the baseline characteristics, intraoperative details, and postoperative outcomes were collected. The baseline characteristics included subject gender, age, ethnicity, preoperative uncorrected visual acuity (UCVA), preoperative best spectacle corrected visual acuity (BSCVA), and preoperative manifest refraction spherical equivalent. The intraoperative details included femtosecond laser settings, pupil tracking ability, anterior chamber gas bubble characteristics and pattern of OBL formation, and the occurrence of any other surgical complications. The postoperative outcomes included UCVA, BSCVA, and manifest refraction spherical equivalent at 2 weeks and 2 months, as well as the occurrence of any other complications during the postoperative period.

### 2.2. Femtosecond Laser Settings

The following laser settings had been programmed for the flap creation in all study subjects: Bed Cut Energy = 0.8 *μ*J, Bed Cut Spot Separation = 8.0 *μ*m, Bed Cut Line Separation = 8.0 *μ*m, Side Cut Energy = 0.8 *μ*J, Side Cut Spot Separation = 5.0 *μ*m, Side Cut Line Separation = 3.0 *μ*m, Vent Canal Power 0.85 *μ*J, and Vent Canal Width = 1.5 mm. All patients had a 9.0 mm flap diameter with a 70° side cut angle. The flap depth ranged from 100 to 110 *μ*m and varied based upon the patient's preoperative corneal thickness measurements.

### 2.3. Statistical Analysis

The JMP 11 mathematical software package from the SAS Institute (Cary, NC, USA) was used to perform the statistical analysis and calculate means with standard deviations. Since the study population is relatively small compared to the frequency of the event being studied, the two-tailed Fisher's exact test was used when comparing the distributions. Results were considered statistically significant at the alpha < 0.05 level.

## 3. Results

A total of 2,886 subject eyes were included in the analysis. The mean age of the overall study population was 37.4 (±11.9) years with 55% female and 45% male. There were a total of 4 eyes of four different patients in which anterior chamber gas bubble formation occurred (incidence = 0.14%). All four cases were females with a mean age of 29.3 (±10.2) years, two of which were Asian and two of which were Caucasian. When comparing Asian eyes (90 total) versus non-Asian eyes (2,796 total) there was a statistically significant difference (*p* = 0.0055). All four patients were myopic, and the mean preoperative refractive spherical equivalent in this small subset of patients was −6.1 (±2.4) diopters, but there was no statistical correlation among formation of anterior chamber gas bubble and the preoperative refractive error (*p* = 0.3063). The mean average keratometry value was 43.8 (±1.7 diopters) for the entire population versus 43.7 (±1.1) diopters for the patients that developed anterior chamber gas bubble, and the mean pachymetry value was 537.9 (±24.6) microns for the entire population versus 539.0 (±16.5) microns for the patients that developed anterior chamber gas bubble formation, neither of which significantly differed (*p* = 0.9955 and *p* = 0.9288, resp.).

**Figure 1:**
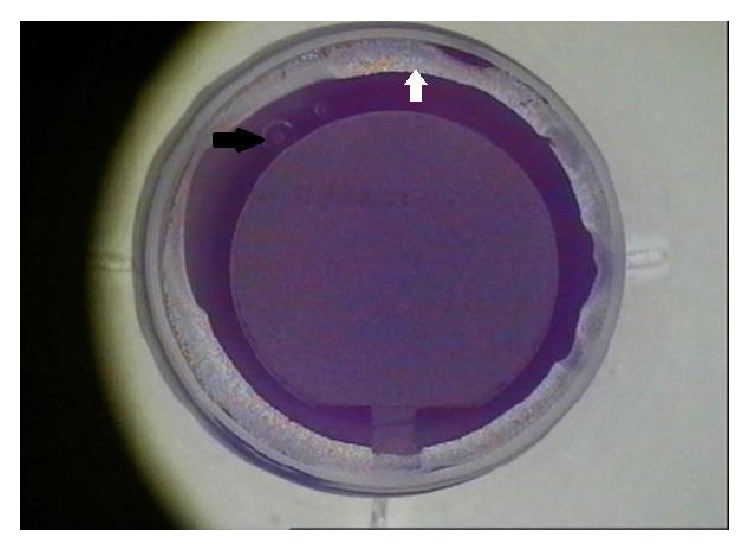
Anterior chamber gas bubble formation during femtosecond laser flap creation for LASIK. Femtosecond laser scout view of the cornea immediately after the femtosecond laser treatment. Note the small anterior chamber bubbles in the upper left-hand corner of the image (black arrow) as well as the 360-degree ring of opaque bubble layer that dissected toward the peripheral cornea until termination at Schwalbe's line (white arrow).

The anterior chamber gas bubble was noted to occur during the lamellar cut in all instances and was immediately preceded by a 360-degree peripheral lamellar ring of deep OBL that dissected near the location of Schwalbe's line (see Figure 1). No cavitation bubbles were noted to evacuate through the venting canal incision in any of these cases. The flap depth treatment was 110 *μ*m in all four instances. The Allegretto Wave Eye-Q 400 Hz excimer laser pupil tracking device was unable to adequately track the pupil so that no immediate attempt was made to lift the flap in any of these patients. One case required postponement of the excimer laser portion of the treatment until the following day, while three cases required a delay until later on during the same day (range: 4–6 hours), two of which still had a solitary miniscule bubble (<0.5 mm) remaining during the excimer laser treatment. All four cases were ultimately able to have successful pupil tracking and excimer laser treatment.

During the postoperative period, there were 72 cases in which the flap was lifted for retreatment due to over- or undercorrection (incidence of 2.49%), 3 cases of flap striae that required refloating of the flap on the first postoperative day (incidence of 0.10%), 2 cases of central toxic keratopathy (incidence of 0.10%), and 1 case of epithelial in-growth (incidence of 0.03%) in the study population. None of these postoperative complications occurred in the subset of patients that experienced anterior chamber gas bubble formation. All eyes that had anterior chamber gas bubble formation during the procedure achieved UCVA of 20/20 postoperatively at 2 weeks and 2 months, and no eyes lost any lines of BSCVA. There were no postoperative infections occurring during the study period.

## 4. Discussion

To our knowledge, this is the first study to evaluate anterior chamber gas bubble formation during femtosecond LASIK. The findings of this study suggest that anterior chamber gas bubble formation may be of little consequence to postoperative refractive outcomes and may not predispose to further complications during or after the completion of the femtosecond LASIK procedure. Nevertheless, formation of anterior chamber gas bubbles is likely to cause delay in the treatment and can potentially lead to increased patient apprehension, inconvenience, and anxiety. In view of the favorable refractive outcomes ultimately achieved in this study, we recommend surgeon patience and patient reassurance in the event of this rare intraoperative occurrence.

Procedural observations during this study lead the investigators to hypothesize that cavitation bubbles dissecting across a lamellar plane in close proximity to Schwalbe's line may, in certain circumstances, gain retrograde access to the anterior chamber through the trabecular meshwork via Schlemm's canal. This hypothesis of trabecular entry has been previously supported with video evidence by Soong and de Melo Franco [12]. Although our overall study population only contained 3.1% Asian eyes, the number of Asian eyes with anterior chamber gas bubble formation during femtosecond laser flap creation was found to be statistically significant (*p* = 0.0055). The authors recognize that an ethnicity correlation with anterior chamber gas bubble formation during femtosecond laser flap creation has not been reported by previous studies and that this correlation must be evaluated by future studies with even larger numbers, particularly in Asian populations, before a valid conclusion can be made. More research is needed to further elucidate both the mechanism of anterior chamber gas bubble formation during femtosecond laser flap creation and specific patient factors that predispose to this infrequent event.

Weaknesses of this study include the retrospective nature of data collection and the limited number of cases for such an evidently rare occurrence. The investigators caution that a different femtosecond laser platform besides the one used in this study, or different laser settings than those used in this study, could have clinically different rates of anterior chamber gas bubble formation as well as postoperative outcomes. Future investigations may validate or refute the findings in this report, further characterize the circumstances in which bubble formation occurs, and investigate various femtosecond laser settings in which the incidence of anterior chamber gas bubble formation during femtosecond laser flap creation is decreased or eliminated altogether in the setting of LASIK.
